# Epichaperomics reveals dysfunctional chaperone protein networks

**DOI:** 10.1038/s41467-023-40713-z

**Published:** 2023-08-22

**Authors:** Mark R. Woodford, Dimitra Bourboulia, Mehdi Mollapour

**Affiliations:** 1 Department of Urology, SUNY Upstate Medical University, Syracuse, NY 13210 USA; 2 Upstate Cancer Center, SUNY Upstate Medical University, Syracuse, NY 13210 USA; 3 Department of Biochemistry and Molecular Biology, SUNY Upstate Medical University, Syracuse, NY 13210 USA

**Keywords:** Systems analysis, Bioinformatics, Networks and systems biology, Chaperones, Cancer genomics

## Abstract

Molecular chaperones establish essential protein-protein interaction networks. Modified versions of these assemblies are generally enriched in certain maladies. A study published in *Nature Communications* used epichaperomics to identify unique changes occurring in chaperone-formed protein networks during mitosis in cancer cells.

‘Chaperome’ is defined as the ensemble of all cellular, molecular chaperones and their regulators, or co-chaperones, that assists the folding of native, intermediates, or misfolded proteins^1,2^. This process safeguards cellular proteostasis^3^. ‘Epichaperomes’ are long-lived heterooligomeric assemblies and disease-associated pathologic scaffolds composed of tightly bound chaperones, co-chaperones, and other factors. They play a key role in many maladies, including cancer and neurodegenerative diseases such as Alzheimer’s, Huntington’s, or Parkinson’s disease^4^. The molecular chaperones heat-shock protein 90 (Hsp90) and heat-shock cognate protein 70 (Hsc70) and their co-chaperones are all essential for epichaperome formation^4,5^. Previous work has identified that 60–70% of tumors express medium-to-high levels of epichaperomes, independent of tissue origin, tumor subtype, or genetic background^3^. However, epichaperome detection and affinity purification, as well as the development of chemical disruptors and quantitation probes, remain a challenge. In this newly published work, Rodina et al. have used their previously reported and refined chemical probes (YK-type)^6^ for targeting and isolating epichaperome-associated Hsp70 (epiHsp70) proteins from cancer cells^7^. Their assessment of 73 cancer cell lines encompassing 9 tumor types, and 19 primary breast tumors, for either vulnerability to YKs or epiHsp70s levels suggest epiHsp70 formation occurs in approximately 70% of tumors. Epichaperomes represent a fraction of the total chaperone pools (approximately 5–35%, depending on the cancer cell line), and thus epiHsp70 is only a small amount when compared to the abundant Hsp70 levels. However, cancers appear to be depending on these epichaperomes for their overall survival and growth^7^.

Within cancer types, there are different levels of epichaperomes. For example, breast cancer MDA-MB-468 and pancreatic cancer ASPC1 cells have comparable Hsp70 levels and other epiHsp70 component chaperones but are differentiated by their epichaperome content, with MDA-MB-468 being epiHsp70s-high and ASPC1 epiHsp70s-low. Interestingly, Rodina et al. also used epiHsp70 epichaperomics to identify thousands of proteins that are primarily involved in the mitotic checkpoint pathway^7^. This dataset also includes the chaperones Hsp70, Hsp90, and Hsp110, the Hsp70 co-chaperone Hsp40, as well as the Hsp90 co-chaperones Aha1 (accelerator of Hsp90 ATPase activity), Cdc37 (kinase clients scaffold) and HOP (decelerator of Hsp90 ATPase) (Fig. 1). The authors further suggest that epiHsp70 proteins not only bind to individual mitotic proteins but also alter the complexation of such proteins. Thus, epiHSP70 may exert its effect on mitosis by altering the complex formation of mitotic checkpoint proteins. These alterations lead to an increase in the fitness of mitotic processes, and while it remains to be seen, perhaps ‘epichaperomes’ serve to promote chromosomal instability and aneuploidy in cancer. Ultimately, this study suggests that unique chaperone networks underlie diverse cellular pathways, and these networks can potentially be specifically interrogated and disrupted using chemical tools built on the YK scaffold.

**Fig. 1:**
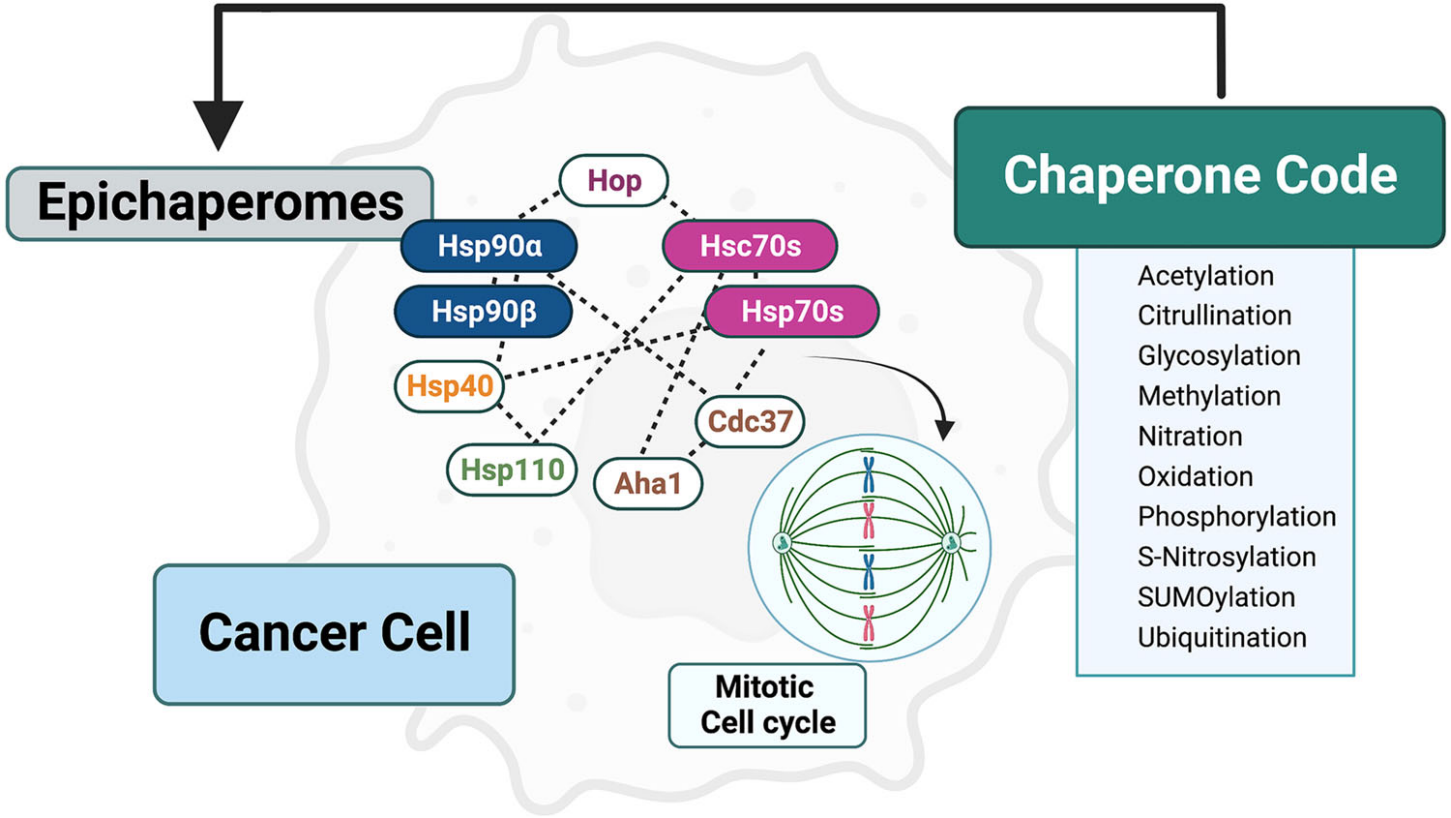
Epichaperomics identifies epichaperomes involved in the fitness of mitotic protein pathways in cancer. Schematic representation of applying epichaperomics to identify protein–protein interaction complexes, also known as epichaperomes, in cancer. epiHsp70s and epiHsp90s consist of heterooligomers that can be detected, isolated, and quantified by several chemical probes. epiHsp70s and epiHsp90s are context-dependent regulators of mitotic cell cycle checkpoints in cancer. An array of post-translational modifications of epiHsp70s and epiHsp90s, also known as the chaperone code, may play a role in the formation of epichaperomes in cancer. Molecular chaperones include Hsp70, Hsc70, Hsp90α, Hsp90β, Hsp40, and Hsp110. Co-chaperones include Hop, Aha1, and Cdc37.

Previous work has shown regulation of the cell cycle through phosphorylation of Hsp70 and Hsp90, also known as the chaperone code^8–11^. Furthermore, emerging clinical data identify Hsp90 inhibition as a promising anti-cancer therapeutic strategy^12^. Cancer cells generally display sensitivity and selectivity towards ATP-competitive Hsp90 inhibitors than their non-tumorigenic counterparts^13,14^. Contributing to this sensitivity is the fact that Hsp90 inhibitors are retained by tumors in vivo, likely as a consequence of Hsp90 binding, far longer than in normal tissues^15^. Mechanistically, it has been previously shown that the mitotic checkpoint kinase Mps1 phosphorylation of Hsp90 sensitizes cancer cells to Hsp90 inhibitors, and elevated Mps1 levels confer tumor selectivity toward amino-domain inhibitors of Hsp90^9^.

Although studies demonstrating the impact of post-translational modifications (PTMs) on the formation of epichaperome complexes are currently lacking, the fact that chaperones and co-chaperones in the chaperome assemblies are post-translationally modified suggests that PTMs will impact the epichaperome assembly. The chaperone code lies at the heart of epichaperome formation, as PTMs (Fig. 1) dictate the chaperome network and chaperone-client interactions^10,11,16,17^. Therefore, the chaperone code may potentially play a major role in dictating epichaperome formation (Fig. 1). The chemical probes evaluated by Rodina et al. to assay the epichaperome can be used to provide valuable information on the impact of PTMs on epichaperome composition and function^7^. These new findings have described the impact of the epichaperome on the mitotic cell cycle, however, further work is necessary to characterize context-specific function and pathway regulation by distinct epichaperome populations. Future studies using epichaperomics will also identify the specific PTMs that are involved in epichaperome formation in a disease context. This will ultimately determine how drugs can effectively target epichaperomes in different maladies, including cancer.
